# Reply to “Stimulus electrodiagnosis and motor and functional evaluations during ulnar nerve recovery”

**DOI:** 10.1590/bjpt-rbf.2014.0181

**Published:** 2016

**Authors:** Daniele Coraci, Federica Porcelli, Valter Santilli, Luca Padua

**Affiliations:** 1 Board of Physical Medicine and Rehabilitation, Department of Orthopaedic Science, “Sapienza” University, Rome, Italy; 2 Don Gnocchi Foundation, Milan, Italy; 3 Physical Medicine and Rehabilitation Unit, Azienda Policlinico Umberto I, Rome, Italy; 4 Department of Geriatrics, Neurosciences and Orthopaedics, Università Cattolica del Sacro Cuore, Rome, Italy

**Keywords:** Chronaxie, ulnar nerve, evaluation studies, disability evaluation, rehabilitation

Dear Editor,

We have read with great attention the paper by Fernandes and colleagues about electrodiagnosis as a monitoring tool in ulnar nerve recovery after surgical treatment of nerve lesion^1^. The study is very interesting and aims to demonstrate that, among the electrodiagnostic parameters, Chronaxie may be the one that best detects the evolution of neuromuscular responses in ulnar nerve recovery. The authors enrolled ten patients who underwent surgical intervention of neurorrhaphy and found a significant reduction in Chronaxie values and a negative significant correlation between Chronaxie and motor function, assessed with the Rosén and Lundborg motor domain score. Given that ulnar nerve lesions are quite common, the importance of the paper is twofold. First, it highlights in the introduction the value of valid diagnostic tools that can correctly evaluate the ulnar nerve lesion, thus allowing physical therapists to plan the best treatment approach. From our point of view, a detailed evaluation of this type of neuropathy is possible through electromyographic assessment and nerve conduction study. These methods allow the evaluation of nerve function and the knowledge of the severity of the lesion^2^. Furthermore, nerve ultrasound can be combined with the previous techniques to visualize the morphological features of the lesion, the exact site of nerve impairment, and the possibilities of anatomical variations^3^ ^,^ ^4^. These data prove crucial to the surgical management specifically tailored for each case. The abovementioned neurophysiological techniques and ultrasound are minimal or non-invasive medical tools that complete the necessary clinical evaluation and together are helpful for diagnosis, prognosis, and treatment approach^5^. The second main point of the paper of Fernandes et al. is the need to find objective methods to assess the recovery of ulnar nerve function after surgical treatment. We consider that, even in this case, needle electromyography can be especially useful, revealing for example the type of voluntary motor unit recruitment in the muscles supplied by the treated nerve. Moreover, ultrasound can reveal the possible evolution of the morphological pattern in comparison with the pre-intervention one^2^. Future studies comparing electrodiagnosis with other neurophysiological and imaging techniques may help us to define the best evaluation method for this type of nerve lesion. A combination of techniques and a comprehensive assessment of the patient, as well as continuous collaboration between physicians and physical therapists, may allow a thorough analysis of the pathological condition and a management strategy tailored to the patient.
