# Supplementary material for: Reply to “Stimulus electrodiagnosis and motor and functional evaluations during ulnar nerve recovery”
Source: Braz J Phys Ther. 2016 May-Jun;20(3):285–6. doi: 10.1590/bjpt-rbf.2014.0181 (PMC4946845; doi:10.1590/bjpt-rbf.2014.0181)
Supplement: Related texts

# Reply: Response to the letter “Stimulus electrodiagnosis and motor and functional evaluations during ulnar nerve recovery”

**Authors:** Luciane F. R. M. Fernandes, Nuno M. L. Oliveira, Danyelle C. S. Pelet, Agnes F. S. Cunha, Marco A. S. Grecco, Luciane A. P. S. Souza

**Affiliations:** 1 Universidade Federal do Triângulo Mineiro (UFTM), Uberaba, MG, Brazil

**Keywords:** Chronaxie, ulnar nerve, evaluation studies, disability evaluation, rehabilitation, movement

Dear Editor and authors of the referred letter,

We thank the authors (Coraci and colleagues) for your interest in our paper and for the great contribution to this important research topic. The authors of the letter demonstrate a good perception of the relevance of our study (Fernandes et al.^1^), which aims to retrieve the use of stimulus electrodiagnosis. Another objective of this paper is to encourage the new generation of physical therapists to study, explore, and use this instrument in clinical practice. In addition, the selection of ulnar nerve lesions was purposeful, due to its high prevalence (Eser et al.^2^).

We understand the value of numerous neurophysiological techniques and nerve ultrasound to guarantee a more complex visualization of the lesion and its progress. We know that these approaches are helpful for diagnosis, prognosis, and treatment of nerve lesions. We opted to use stimulus electrodiagnosis because it is the only instrument that physical therapists can use in clinics. We can receive the results of the other techniques, but we cannot conduct evaluations such as ultrasound and electroneuromyography. Therefore, we tried to reinforce the importance of stimulus electrodiagnosis as a physical therapy tool, which obviously has to be combined with the results of other exams. We agree with the authors of the letter that this broad approach can contribute to the correct diagnosis and to a better follow-up of all nerve lesions.

The impact of future studies on this area, as suggested by the authors of the letter, can promote significant changes in clinical practice. We believe that each technique has its value, and all techniques can point out important and different aspects of the lesion and its recovery. We also agree that collaboration between physicians and physical therapists might allow a comprehensive appraisal of the pathological condition and a correct management tailored to the patient.

We are grateful for our opportunity, as physical therapists, to reflect on our approaches and on the combination of methods of evaluation in order to attain a better understanding of nerve lesions and the recovery of patients.

## References

[1] Fernandes LFRM, Oliveira NML, Pelet DCS, Cunha AFS, Grecco MAS, Souza LAPS (2016). Stimulus electrodiagnosis and motor and functional evaluations
during ulnar nerve recovery. Braz J Phys Ther.

[2] Eser F, Aktekin LA, Bodur H, Atan C (2009). Etiological factors of traumatic peripheral nerve
injuries. Neurol India.
